# Green light activated hydrogen sensing of nanocrystalline composite ZnO-In_2_O_3_ films at room temperature

**DOI:** 10.1038/s41598-017-12547-5

**Published:** 2017-09-22

**Authors:** A. S. Ilin, M. I. Ikim, P. A. Forsh, T. V. Belysheva, M. N. Martyshov, P. K. Kashkarov, L. I. Trakhtenberg

**Affiliations:** 1 Physics Department, Lomonosov Moscow State University, Leninskie Gory 1-2, Moscow, 119991 Russia; 2 Semenov Institute of Chemical Physics, Russian Academia of Sciences, 4, Kosygina Str., Moscow, 119991 Russia; 3 National Research Centre “Kurchatov Institute”, 1, Akademika Kurchatova pl., Moscow, 123182 Russia; 4 Moscow Institute of Physics and Technology (State University), 9, Institutskii per., Dolgoprudny, Moscow, Region 141700 Russia

## Abstract

The possibility of reducing the operating temperature of H_2_ gas sensor based on ZnO-In_2_O_3_ down to room temperature under green illumination is shown. It is found that sensitivity of ZnO-In_2_O_3_ composite to H_2_ nonmonotonically depends on the oxides’ content. The optimal ratio between the components is chosen. The new mechanism of nanocrystalline ZnO-In_2_O_3_ sensor sensitivity to H_2_ under illumination by green light is proposed. The mechanism considers the illumination turns the composite into nonequilibrium state and the photoconductivity change in the H_2_ atmosphere is linked with alteration of nonequilibrium charge carriers recombination rate.

## Introduction

Nanocrystalline metal oxides are widely investigated as sensitive materials for gas sensors working at high temperature (300–500 °С). High operating temperature leads not only to high power consumption but also to the fire and explosion risks and impossibility of gas sensor embedding in the electronic boards of mobile devices. In this regard, considerable research efforts have been devoted to reduction of the gas sensor operating temperature.

The effect of molecules adsorption on photoconductivity of semiconductors was known a long time^1,2^ ago but investigations of the influence of illumination on sensor response began only in the last decade. Most of the studies are devoted to the influence of ultraviolet (UV) illumination on the sensor properties of nanocrystalline metal oxide to oxidizing gases^3–6^. However UV light emitting diodes (LEDs) have lower efficiency and are much more expensive than visible LEDs. Therefore the utilizing of visible LEDs for the sensor response activation is more effective from a practical point of view.

Much less attention is paid to the effects of visible illumination and mainly effects of irradiation on response to gases-oxidizing agents are investigated. Number of researches studying the photo-activation of the sensor response to reducing gases is much smaller, and there is no definitive interpretation of the observed processes (see, e.g.^7–10^). In addition, proposed explanations of the illumination effect on the sensor response of metal oxides (to both oxidizing and reducing gases) don’t consider that the illumination realizes non-equilibrium condition. Therefore, the description of the electronic processes under the illumination should be in terms of photoconductivity instead of conductivity. Photoconductivity, in its turn, strongly depends on the charge carriers recombination and generation. And the observed effects of gases adsorption on the surface of metal oxides under illumination can be determined by generation-recombination processes.

As early reported, metal oxides composites^11–15^ and particularly ZnO-In_2_O_3_ composites^16–19^ exhibit the enhanced gas sensitivity to reducing gases. However, the effect of visible illumination on the sensitivity of ZnO-In_2_O_3_ composites to reducing gases was not investigated.

Effect of green light irradiation on gas sensing behavior of the hydrogen sensors based on the nanocrystalline ZnO-In_2_O_3_ composites is firstly reported. The dependence of photoactivated sensor response to H_2_ on the fraction of ZnO in ZnO-In_2_O_3_ composite is analyzed. A new mechanism of the sensor response under illumination is proposed, which considers the transition of composite to nonequilibrium state under the light irradiation.

## Results and Discussion

**Figure 1:**
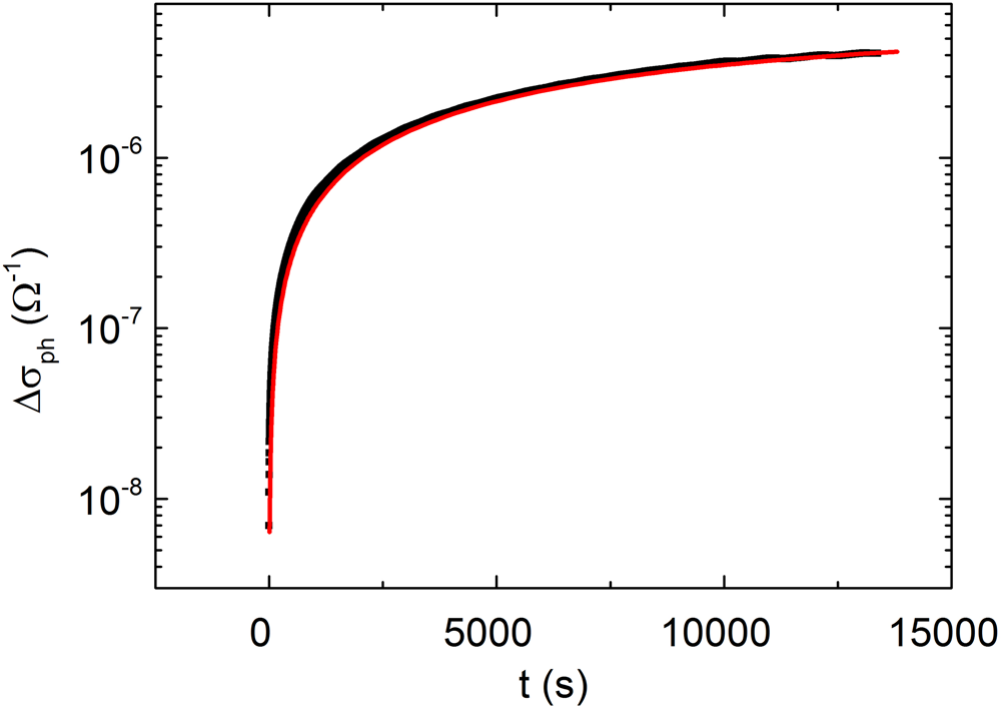
Transient photoconductivity of composite ZnO-In_2_O_3_ film containing 65 wt.% ZnO. The approximated parameters *σ*_*ph*_ = 5.67∙10^−6^ Ω^−1^ and τ = 10200 s according to equation (1).

The kinetic of photoconductivity increase of ZnO-In_2_O_3_ composite film containing 65 wt.% ZnO is presented in Fig. 1 (wt.% - relative mass fraction).

The similar dependences have been obtained for other samples. The photoconductivity kinetic for all samples can be approximated by exponential dependence

1 $${\rm{\Delta }}{{\rm{\sigma }}}_{ph}(t)={{\rm{\sigma }}}_{ph}(1-{e}^{-t/\tau })$$

where, according to^20^, ${\sigma }_{ph}$ – stationary photoconductivity and $\tau$ – relaxation time of photoconductivity.

Excitation of nonequilibrium carriers under illumination should be monopolar, as the energy of green light photons is smaller than band gap values of ZnO and In_2_O_3_^21–23^. Nonequilibrium charge carriers can be excited from either localized states in the band gap or surface states, which are presented in nanocrystalline ZnO and In_2_O_3_ in high concentrations. These states may be shallow and become ionized easily under the green illumination. For example, the existence of shallow traps (about 1 eV below the conduction band) was shown in nanocrystalline In_2_O_3_^24^.

**Figure 2:**
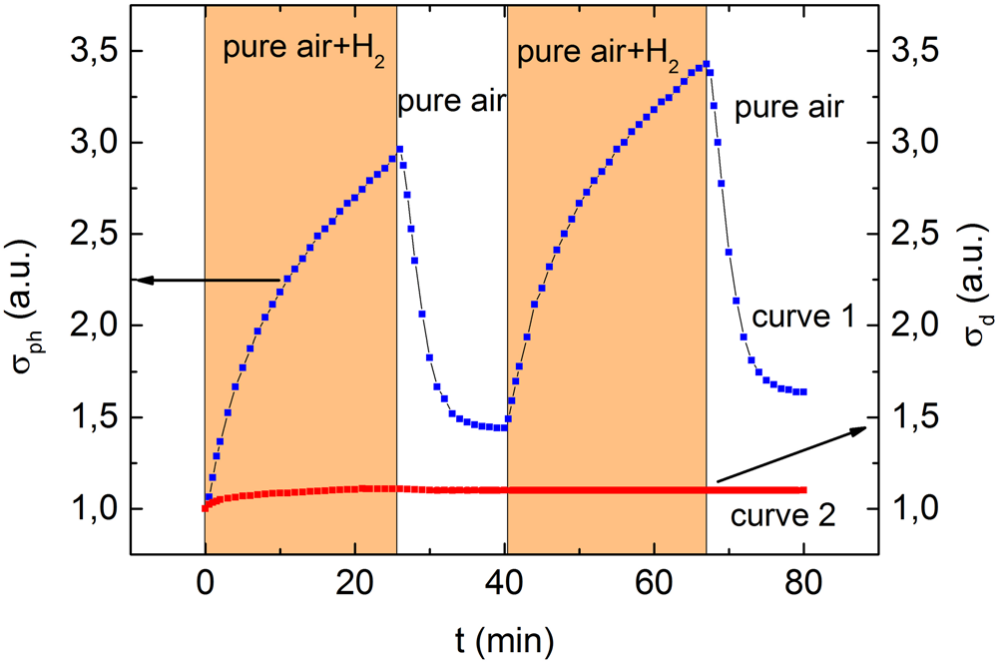
Photoconductivity (curve 1) and conductivity (curve 2) alteration of ZnO-In_2_O_3_ composite film containing 10 wt.% ZnO under periodic exposure to air containing hydrogen.

The gas sensing of composite films under the illumination was determined by the following procedure. Firstly, the samples were illuminated for several hours while the photoconductivity reached the steady state. Afterwards the mixture of pure air and hydrogen was supplied into the chamber for 25 minutes and then the pure air flowed through the chamber for the sample recovery. The cycle of exposure of sample to air with hydrogen and pure air was repeated to demonstrate the reproducibility of the results. The photoconductivity alteration of composite ZnO-In_2_O_3_ film containing 10 wt.% ZnO during exposure to air with hydrogen and pure air is shown in Fig. 2 (curve 1).

To facilitate the analysis the photoconductivity in Fig. 2 is normalized to the value of stationary photoconductivity in the pure air. One can see that the photoconductivity varies greatly in the atmosphere with hydrogen. For comparison the dark conductivity of the samples exposed to air with hydrogen are also shown in Fig. 2 (curve 2). Also, as in the case of photoconductivity, dark conductivity is normalized by the conductivity of sample in the pure air. It is seen that dark conductivity practically doesn’t depend on the hydrogen content in the atmosphere. The similar results have been obtained for other samples. The exposure of all samples to hydrogen results in photoconductivity alteration while the dark conductivity remains almost constant.

According to^20,25^, the stationary photoconductivity is determined by the relaxation time $\tau$, mobility $\mu$ and generation rate of electrons $g$ (for monopolar excitation):

2 $${{\rm{\sigma }}}_{ph}=e\mu g\tau $$

The relaxation time of photoconductivity is determined by the processes of the electrons recombination in the sample. Since the kinetics of photoconductivity for all samples is described by a simple exponential dependence (equation (1)), the relaxation time of photoconductivity $\tau$ does not depend on the concentration of electrons and is determined only by the location and concentration of recombination centers.

In nanocrystalline and polycrystalline materials recombination centers are often assumed to be located at the boundaries of the nanocrystals^26,27^. In our samples the defects like dangling bonds (indium and zinc vacancies)^21,27–29^ and oxygen molecules can be such recombination centers. In our previous work^23^ we showed that the photoconductivity of nanocrystalline indium oxide strongly depends on the oxygen molecules adsorbed on the surface.

The photoconductivity increases in the air with hydrogen. As the parameters of illumination don’t change, it is reasonable to assume that the photoconductivity varies due to the alteration of relaxation time of photoconductivity and mobility of electrons. The concentration of recombination centers decreases significantly in the atmosphere of hydrogen. The hydrogen may passivate the defects by incorporating in the zinc and indium vacancies on the surface^30–32^. In addition, the oxygen molecules adsorbed on the surface of oxides can form a chemical bond with hydrogen and desorb^33,34^. The concentration of recombination centers decrease results in increase of photoconductivity relaxation time and, in accordance to equation (2), stationary photoconductivity in the hydrogen atmosphere increases as observed in experiment.

In the atmosphere of hydrogen without illumination the defect passivation and decrease of the adsorbed oxygen concentration take place too. However, the dark conductivity is not determined by recombination processes, but depends mainly on the concentration of free electrons and their mobility. The fact that the dark conductivity does not change in the atmosphere of the hydrogen indicates that the carrier mobility does not change. As the mobility under illumination and in the dark, as a rule, do not differ from each other, we can assume that the observed changes of photoconductivity are not associated with a change in mobility.

Thus, we can assume that the change of photoconductivity in hydrogen atmosphere is determined by a significant change of the charge carriers recombination rate. It should be noted that the desorption of oxygen can result in increase of the electrons concentration (during the desorption oxygen gives up an electron to the conduction band^35^). This electrons release can increase the dark conductivity. However, initially in nanocrystalline In_2_O_3_ and ZnO (in the pure atmosphere before hydrogen adding) free charge carriers concentration is very high^21,22^ (because of the large number of oxygen vacancies inside the nanocrystals) and the change in concentration during the hydrogen adsorption is negligible. Therefore, the electron concentration change in the air with hydrogen at room temperature practically does not affect the value of the dark conductivity, which leads to the absence of gas response.

In order to assess how the alteration of photoconductivity in hydrogen depends on the relative fraction of ZnO in the composite ZnO we estimated the sensor response to hydrogen. The sensor response $S$ was determined as:

3 $$S=\frac{{\sigma }_{ph}({H}_{2})}{{\sigma }_{ph}(air)}$$

where ${\sigma }_{ph}({H}_{2})$ and ${\sigma }_{ph}(air)$ – stationary photoconductivity of sample in the hydrogen atmosphere and in pure atmosphere, respectively.

**Figure 3:**
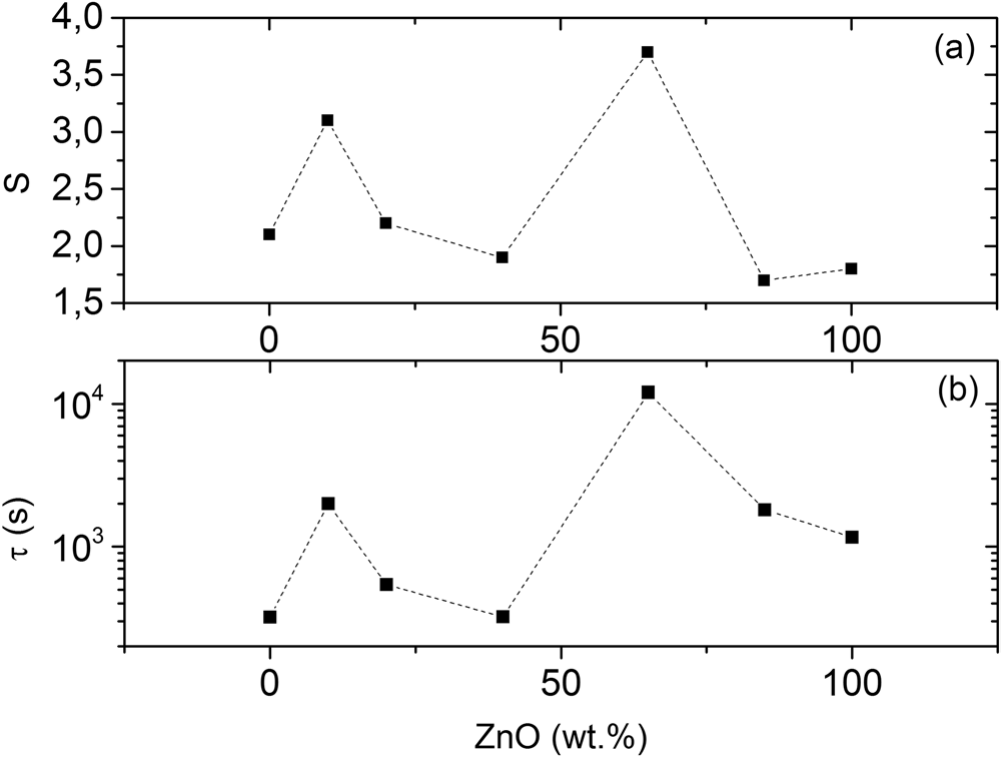
Sensor response to H_2_ (**a**) and photoconductivity relaxation time (**b**) of composites ZnO-In_2_O_3_ with different relative fraction of ZnO.

Figure 3a shows the sensor response dependence on the relative fraction of ZnO in the composite ZnO-In_2_O_3_. It is seen that the sensor response depends on the ZnO concentration non-monotonically. Figure 3b shows the relaxation time of photoconductivity dependence on the relative fraction of ZnO in the composite ZnO-In_2_O_3_. There is a good correlation between the relaxation time of photoconductivity and sensor response, namely the increase of sensor response is accompanied by increase of the relaxation time of photoconductivity. This fact confirms the assumption that changes of photoconductivity in hydrogen atmosphere in the composites ZnO-In_2_O_3_ are determined by the recombination.

The relaxation time of photoconductivity in the composites of ZnO- In_2_O_3_ may be determined by various processes. First, the recombination centers in ZnO and In_2_O_3_ have different nature and different parameters of the carriers’ capture. The concentration change of one kind of centers leads to more significant change in the photoconductivity relaxation time than the concentration change of other centers does. Second, there is a spatial separation of electrons and holes in the ZnO-In_2_O_3_ composites. According to^36,37^, in the energy diagram of heterojunction ZnO-In_2_O_3_ ZnO is a potential well for electrons, and In_2_O_3_ – potential well for holes. The separation of charges increases the relaxation time of photoconductivity, especially this effect should be noticeable for composites with approximately same content of In_2_O_3_ and ZnO. Third, the ZnO nanocrystals are less sensitive to hydrogen than In_2_O_3_ nanocrystals^38^. It can explain the observed small difference in the dependence of the sensor response and the photoconductivity relaxation time for pure ZnO in Fig. 3.

The competition of these processes may explain the non-monotonic dependence of the photoconductivity relaxation time on the relative fraction of ZnO in the composite ZnO- In_2_O_3_ (see Fig. 3b). At the same time, it can be assumed that the recombination rate of nonequilibrium electrons is relatively small for the ZnO- In_2_O_3_ composites with large values of τ before gas injection. Since the hydrogen concentration in the atmosphere with H_2_ is the same for all samples, the change in the recombination centers concentration is assumed to be the same. The last in turn affects the recombination rate. The recombination rate change should be more significant in relative units for samples with initially low recombination rate and ratio of photoconductivity in atmosphere of hydrogen to initial photoconductivity in pure air for these samples should be greater. It explains the observed correlation between sensor response and photoconductivity relaxation time.

The dependence of the sensor response on relative fraction of ZnO in the ZnO-In_2_O_3_ composite allows to determinate the optimal composition for H_2_ detection under illumination. According to Fig. 3 the optimal content of ZnO in the ZnO-In_2_O_3_ composite should be near 60 wt.%. As was shown earlier, at high working temperature (400 °C) the highest response to H_2_ was observed for ZnO-In_2_O_3_ composites containing 20 wt.% and 80 wt.% ZnO^16^. The difference between the relative fractions of ZnO in the composite, at which the maximum gas response was observed, confirms different H_2_ sensitive properties of the composites under illumination and heating.

In conclusion, we have shown the possibility of reducing the operating temperature of H_2_ gas sensor based on ZnO-In_2_O_3_ down to room temperature under green illumination. It is found that sensitivity of ZnO-In_2_O_3_ composite to H_2_ nonmonotonically depends on the oxides’ content, which allows to choose the optimal ratio between the components to achieve the highest sensitivity. The maximum sensitivity to 970 ppm of H_2_ is observed for the nanocomposite films based on 65 wt.% ZnO and 35 wt.% In_2_O_3_. It should be noted that the studied composites did not exhibit the sensitivity to hydrogen at room temperature without illumination.

We proposed the new mechanism of nanocrystalline ZnO-In_2_O_3_ sensor sensitivity to H_2_ under illumination by green light. The mechanism considers the illumination turns the composite into nonequilibrium state. In this regard the photoconductivity of the ZnO- In_2_O_3_ composite change in the H_2_ atmosphere is largely determined by recombination processes. The recombination processes determine the photoconductivity relaxation time which in turn determines the photoconductivity of the composite. It is shown that the photoconductivity relaxation time is linked with the response of the ZnO-In_2_O_3_ composites to H_2_. The possible recombination mechanisms of non-equilibrium charge carriers in the studied structures are discussed. The proposed mechanism links the response of the ZnO-In_2_O_3_ composites to H_2_ under illumination with change of the nonequilibrium charge carriers recombination rate.

## Methods

Nanocrystalline composite films were synthesized from commercial nanopowders In_2_O_3_ (AnalaR grade, 99.5%, BDH/Merck Ltd., Lutterworth, Leicestershire, UK) and ZnO (99.9%, Sigma–Aldrich Chemical Co., Gillingham, UK). The average nanocrystal size of the oxides in the original powder is 50–80 nm.

Nanocrystalline composite films were prepared using aqueous suspensions of nanopowders. The certain amounts of ZnO and In_2_O_3_ were mixed and triturated in an agate mortar with a small amount of distilled water until a homogeneous suspension with pre-determined mass proportion of constituent oxides was obtained. The resulting slurry was screen printed on a dielectric alumina substrate equipped with platinum contacts and a heater. The resulting layer was heated during 3 hours at 120 °C and then annealed in air, the temperature was gradually raising up to 550 °C and was kept at this value until a constant resistance value of the resulting nanocomposite film was obtained.

The structure and particle size were examined by transmission electron microscopy using the device JEM-2100 (Jeol, Japan). Detailed analysis was described elsewhere^16^. The resulting nanocomposite films are a mixture of nanocrystals of the corresponding oxides with variety of shapes and wide particle size distribution from 40 to 80 nm, which practically coincides with the sizes of the original commercial nanopowders claimed by the manufacturers.

**Figure 4:**
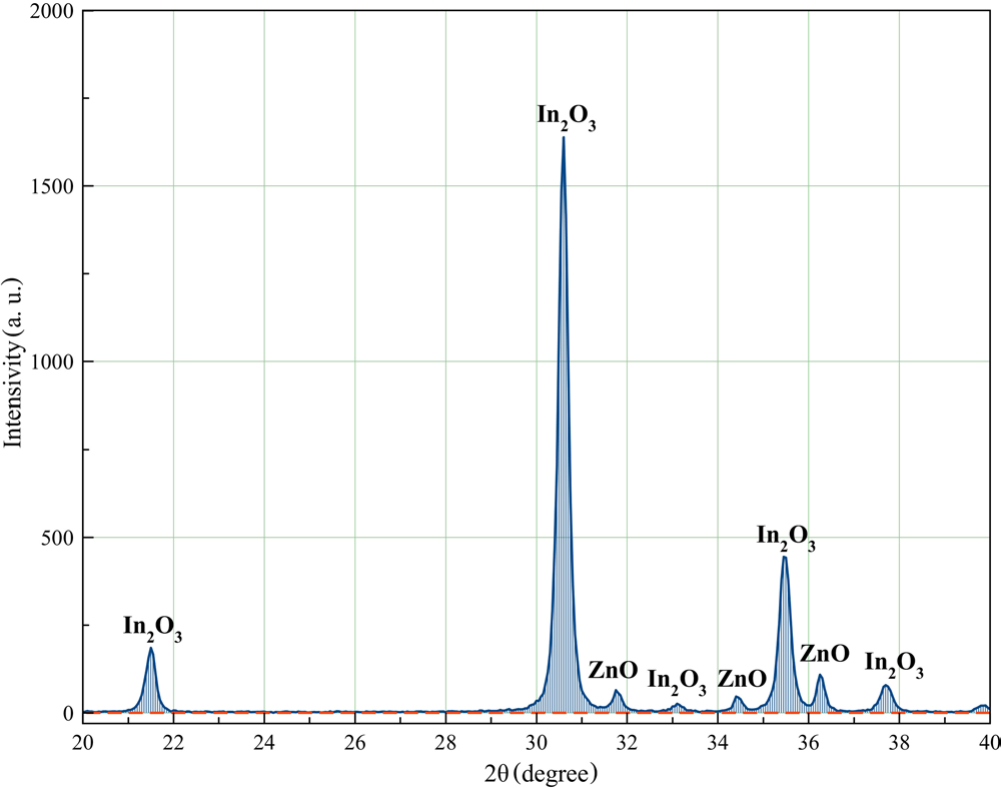
XRD spectra of ZnO-In_2_O_3_ composite film containing 10 wt.% ZnO.

The phase composition of the metal oxide films was determined by X-ray diffraction on Ultima IV (CuKα1 (X-ray wavelength *λ*_Cu_ = 0.154056 nm)). A typical X-ray diffraction pattern of the nanocomposite metal oxide film ZnO-In_2_O_3_ is shown in Fig. 4. It is shown that the composition of the resulting film coincides with the composition of the initial mixture of metal oxides.

Experiments were carried out at room temperature. Illumination of the samples was performed by green LED with a maximum intensity at 525 nm. The intensity of light incident on the sample was 5 mW/cm^2^. The photoconductivity ${\rm{\Delta }}{\sigma }_{ph}$ was determined as difference between conductivity under illumination ${\sigma }_{ill}$ and dark conductivity *σ*_*d*_:

4 $${\rm{\Delta }}{\sigma }_{ph}={\sigma }_{ill}-{\sigma }_{d}$$

For sensor properties investigation the samples were placed in chamber with mounted LED and pure air or air containing 970 ppm of hydrogen flowed through the chamber.

The datasets analysed during the current study are available from the corresponding author on reasonable request.
